# Ultrastructural changes in the fat body of desert locust, *Schistocerca gregaria* (Orthoptera: Acrididae) treated with zinc chromium oxide nanostructures via chemical co-precipitation approach

**DOI:** 10.1186/s13065-023-00914-5

**Published:** 2023-02-20

**Authors:** Fatma M. Hashem, Elsayed Elgazzar, Wageha A. Mostafa

**Affiliations:** 1 Entomology Section, Zoology Department, Faculty of Science, Zagazig University, Zagazig, 44519 Egypt; 2 Department of Physics, Faculty of Science, Suez Canal University, Ismailia, 41522 Egypt

**Keywords:** Ultrastructure, Fat body, *Schistocerca gregaria*, ZnCrO NPs, Nanospheres, Energy gap

## Abstract

The present work aims to investigate the ultrastructural changes in the fat body of fifth instar nymphs *Schistocerca gregaria* (Orthoptera: Acrididae) treated with zinc chromium oxide (ZnCrO). The nanoparticles (NPs) were prepared by co-precipitation route and characterized using X-ray diffraction (XRD), energy dispersive X-ray spectroscopy (EDX), scanning electron microscopy (SEM), and transmission electron microscopy (TEM). The ZnCrO NPs exhibited polycrystalline hexagonal structure, composed of spherical–hexagonal shapes with an average size ~ 25 nm. Besides, the UV–Vis spectrophotometer (Jasco-V-570) was utilized for optical measurements. The energy gap $\left( {{\text{E}}_{{\text{g}}} } \right)$ was estimated from the transmittance (T%) and reflectance (R%) spectra through the range of 3.307–3.840 eV. In biological sections, *S. gregaria* 5th instar nymphs, TEM images demonstrated that the fat body was strongly impacted with the concentration 2 mg NPs result in great agglomeration of chromatin in the nucleus as well as haemoglobin cells (HGCs) pierced with malformed trachea (Tr) at 5th and 7th days post treatment. The obtained results indicated a positive action of the prepared nanomaterial on *Schistocerca gregaria* fat body organelles.

**Graphical Abstract:**

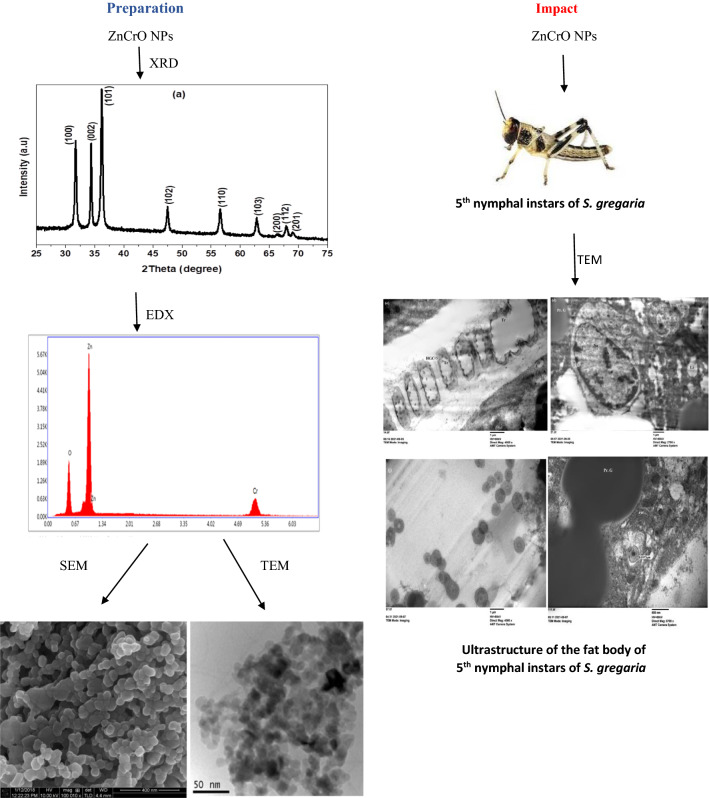

## Introduction

Nanotechnology is considered one of the modern and attractive fields owing to its advantages and wide range applications extend from chemical engineering and communication systems to biotechnological activities. Currently, nanomaterials are utilized in agriculture and expected to revolutionize the field of pest management in the near future. The importance of this technology has emerged in offering novel ways for designing active ingredients and crop protection [1–3]. Several studies have used the nanoparticles (NPs) to replace traditional chemical pesticides against weeds, plants pathogens, and insect pests dependence of their safety toward the environment and human beings [4]. Unlike conventional hydrophobic pesticides, nanocides may be water-soluble (hydrophilic) which enhances bioactivity and coverage uniformity. Because they can be applied in small volumes and taken up quickly by cells, the use of nanocides can slow the development of resistance in target pests [5–7]. Recently, metal oxide based nanoparticles, such as magnesium oxide (MgO), tin oxide (SnO_2_), titanium dioxide (TiO_2_), silver oxide (Ag_2_O), and zinc oxide (ZnO) have been used in biosensors, catalysis, biomedical imaging, optoelectronic devices, and medicine industry. Specifically, ZnO nanostructures of low toxicity level, small crystallite size, high chemical reactivity, and good chemical stability can be used in biological fields without hazardous beneficial organisms [8]. Keerthana et al. [9] have reported the distinctive aspects of ZnO NPs in the agricultural field as biogenesis for protecting yield food crops and improving the growth of *Abelmoschus esculentus* in a safe and nontoxic regime. However, promoting of structural and physical characteristics of ZnO by doping with appropriate transition metal like Cu, Mn, Fe, Co, Ag, Ru, Pd, etc. have important role in many of technological areas. Among the integrated possibilities, inducing chromium (Cr) ions into ZnO framework revealed great advantages owing to the small ionic radius of chromium ions (0.062 nm) compared with zinc ions (0.074 nm) as well as their similar electronegativity values [10]. These aspects enable Cr^3+^ to be easily incorporated inside the host ZnO lattice structure producing new energy levels inside the band gap, increasing of free charge and transport density. Debnath et al. [11] have improved the physical properties of pure ZnO by chromium ions synthesized by chemical approach. It is worth noting that integrating Cr3+ inside ZnO leads to increase active sites and surface to volume area [11]. The desert locust, *Schistocerca gregaria* [12–14] is a species of locust, a periodically swarming, short-horned grasshopper in the family Acrididae. They are found primarily in the deserts and dry areas of northern and eastern Africa, Arabia, and southwest Asia. The desert locust shows periodic changes in its body form and can change in response to environmental conditions, over several generations, from a solitary, shorter-winged, highly fecund, non-migratory form to a gregarious, long-winged, and migratory phase in which they may travel long distances into new areas. In some years, they may thus form locust plagues, invading new areas, where they may consume all vegetation including crops, and at other times, they may live unnoticed in small numbers. The desert locust’s migratory nature and capacity for rapid population growth present major challenges for control, particularly in remote semiarid areas, which characterize much of their range [15]. The fat body of *Schistocerca gregaria* is an important target of toxic metals and is involved in multiple homeostatic functions regulating nutrient synthesis and storage or providing several metabolic pathways [16, 17]. The peripheral fat body of *Schistocerca gregaria* (Orthoptera: Acrididae) occupies the peripheral portion, which presents an anatomical relation showing a delicate granular, long striped shape, irregular distribution reaching the anterior portion of the prothorax [18]. Fat bodies have important roles in detoxification, endocrinology, reproduction and nutrition, so damage to this organ may indicate relevant sublethal effects that may comprise insect development and behavior [19, 20]. Nanopesticides that penetrate the organisms of insects leading to mortality have attracted much attention throughout the current decade [20, 21]. Nanometal oxides can be developed by using various chemical and physical techniques such as hydrothermal microwave irradiation, combustion synthesis, magnetron sputtering, sol–gel, and electrochemical methods [22, 23]. The current study was undertaken to synthesize and characterize ZnCrO NPs by chemical co-precipitation reaction and to study its impact on the fat body of *S. gregaria* fifth instar nymphs.

## Experimental section

### Materials and methods

#### Preparation and characterization of zinc chromium oxide nanopowder

Zn_1−x_Cr_x_O nanopowder (x = 10 wt%) was prepared by a cost-effective chemical co-precipitation. In this chemical reaction, 12.15 g zinc acetate dihydrate [Zn(CH_3_COO)_2_·2H_2_O] was dissolved in 55 ml deionized water and 2.65 g chromium acetate dihydrate [Cr_2_(OOCCH_3_)_4_·2H_2_O] in 20 ml using magnetic stirrer. Chromium acetate was slowly added to zinc acetate solution with continuous stirring for 2 h at the room temperature. Then, 4.15 g sodium hydroxide (NaOH) was dissolved in 55 ml deionized water and gradually added dropwise to aqueous zinc/chromium acetate solution with stirring at constant speed (900 rpm/min) for 5 h. A homogeneous white gray precipitate powder was formed at pH ~ 9. After that, the obtained powder was washed, dried at 80 °C for 12 h and eventually calcined at 450 °C for 3 h.

#### Characterization of the nanomaterial

The prepared ZnCrO was characterized by XRD (Rigaku Smart Lab.) at wavelength λ = 1.54 Å (Cu Kα radiation) with 2θ angle changed from 25° to 75°. Energy dispersive X-ray analysis (EDX) attached with scanning electron micrograph (SEM; Helios Nanolab. 400) were examined to identify the element composition and surface morphology. Before the SEM technique, the sample was bombarded with evaporated conductive iridium (Ir) atoms via a spin coater sputter coater (Model, EMS 150T ES). To identify the mean size and particle distribution, transmission electron microscopy (TEM; Hitachi-H-7500) was employed at 100 kV. The UV–Vis spectrophotometer JASCO (V-570) was utilized for measuring transmittance and reflectance spectra and determination of the type of energy gap transition. The thin films were prepared by dissolving 5 mg of ZnCrO NPs into 15 ml ethanol/dimethylformamide (DMF). The solution was deposited drop by drop on the glass substrates using spin coating technique. Thereafter, the fabricated thin films were left for drying in a furnace at 200 °C for 1 h.

#### Insect rearing

Individuals of the desert locust, *S. gregaria* were obtained from the Plant Protection Research Institute, Zagazig, Sharkia Governorate, Egypt. Adults were reared in the laboratory under crowded conditions at 30 ± 2 °C, 70–80% R. H. and a photoperiod of 8 D: 16 L for several generations. Adults were placed in wooden-framed cages measuring 40 × 40 × 60 cm as described by [24, 25].

#### Treatment

The experimental design was arranged with four groups of treatments and three replicates, and each experiment consisted of ten 5th instar nymphs of *S. gregaria*. The first group was sprayed with 0.150 mg of zinc chromium oxide NPs, the second group was sprayed with 1 mg of zinc chromium oxide, the third group was sprayed with 2 mg of zinc chromium oxide and the fourth group was a control.

#### Electron microscope studies

Nymphal instars from the 4 tested groups were prepared for electron microscopy by anesthetizing them with CO_2_ and then killing them by twisting the head to break the “neck” membrane and collecting the peripheral fat body of the thorax at intervals of 3, 5 and 7 days post treatment which was immobilized by chilling for a few minutes on ice.

They were dissected in ice- cold Ringer’s solution (86 ml M NaCl, 5.4 ml M KCl, 3 ml MCaCl_2_ × 2H_2_O containing a small amount of phenylthiourea) and transferred to a fixative consisting of 2.5% glutaraldehyde (Sigma) in 0.1 M cacodylate buffer (pH 7.2) for approximately 10 min, dissected free and put into fresh fixative for 1–2 h. after washing in 0.1 M cacodylate buffer, the specimens were postfixed in 1% OsO4 in the same buffer for 1 h, washed, dehydrated in an ethanol series, and embedded in Araldite epoxy resin.

Semithin sections for light microscopy and ultrathin sections for electron microscopy (EM) were cut on a Leica EM KMR2 ultramicrotome. The Semithin sections were stained with toluidine blue, meanwhile Ultrathin sections (80 nm) were stained with 4% uranyl acetate and 0.4% lead citrate in distilled water and examined with a JEOL 1200 EX II transmission electron microscope at the central laboratory, Faculty of Science, Zagazig University.

### Results and discussion

#### Crystallographic, elemental composition, and microstructure analysis of Zn_1−x_Cr_x_O NPs

**Fig. 1:**
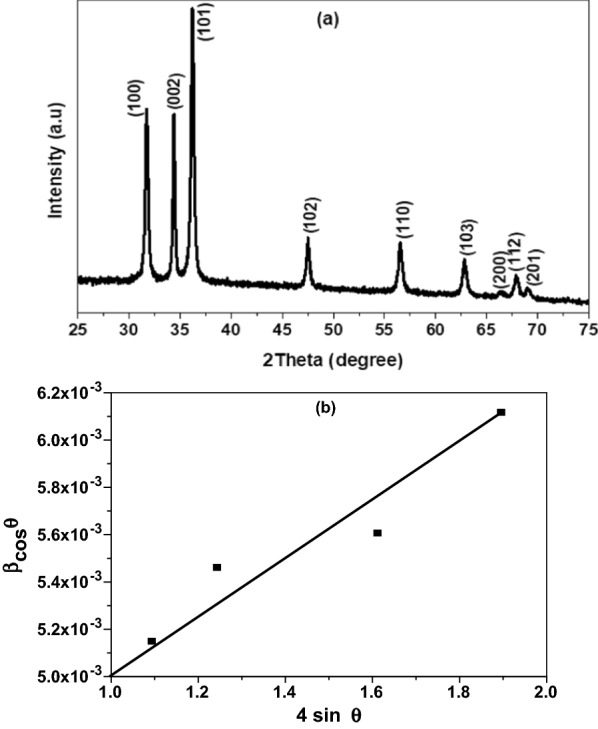
**a** XRD pattern of ZnCrO and **b** W–H method of Cr doped ZnO nanopowder annealed at 450 °C

Figure 1a illustrated the XRD pattern of Cr-doped ZnO annealed at 450 °C for 3 h. As shown, the nanopowder of polycrystalline structure nature with a wurtzite hexagonal phase and matching with the standard XRD data (JCPDS card no: 36-1451, space group P63mc) [26, 27]. The sharp peaks distinguished by high intensity revealing the high crystallinity degree. No extra peaks attributed to hydroxyl (–OH) groups, chromium oxide (Cr_2_O_3_) or chromium metal ions Cr^3+^ are observed in the pattern, which emphasize the purity of the nanostructure [27, 28]. Additionally, the close electronegativity values of Zn (1.65) and Cr (1.65) as well as the small ionic radius of chromium ions (0.062 nm) led to Cr^3+^ easily incorporated into the ZnO lattice [29, 30]. The crystallite size $\left( {\text{D}} \right)$ of the nanoparticles was evaluated from the Scherer formula and confirmed by the Williamson–Hall (W–H) method using the following equations [31, 32]:

1 $${\text{D}} = \frac{{{\text{K}}\uplambda }}{{\upbeta \cos\uptheta }},$$

2 $$\upbeta \cos\uptheta = \frac{{{\text{k}}\uplambda }}{{\text{D}}} + 4\upvarepsilon \sin {\uptheta ,}$$

where *λ* is the X-ray wavelength equals 1.540 Å for Cu Kα, k is the shape factor of constant value equals 0.94, $\upbeta$ is the FWHM representing instrumental broadening, ${\uptheta }$ is Bragg’s scattering angle, and ${\upvarepsilon }$ is the lattice strain. The Scherer equation was applied on the strongest peak of the preferred, Miller indices (hkl 101) which used to determine the planes and lattice dimensions. Moreover, the Williamson–Hall (W–H) equation was defined from $\upbeta \cos\uptheta \;{\text{to}}\;4\sin\uptheta$ plot, illustrated in Fig. 1b. The results obtained from W–H method are more accurate compared to Scherer equation in which both crystallite size and the influence of strain are involved through the XRD planes [31, 33]. As demonstrated in Fig. 1b, the strain $(\varepsilon )$ was determined from the slope and the crystallite size $\left( {\text{D}} \right)$ from the intercept $\left( {\frac{{{\text{k}}\uplambda }}{{\text{D}}}} \right)$ by knowing the values of k and $\uplambda$. The dislocation density $\left(\updelta \right)$ was evaluated by the relation [32, 33]:

3 $$\updelta = \frac{1}{{{\text{D}}^{2} }},$$

and the specific surface area $\left( {{\text{S}}_{{\text{a}}} } \right)$ was given by [31]:

4 $${\text{S}}_{{\text{a}}} = \frac{6}{{{\text{D}} \times\uprho }}.$$

**Fig. 2:**
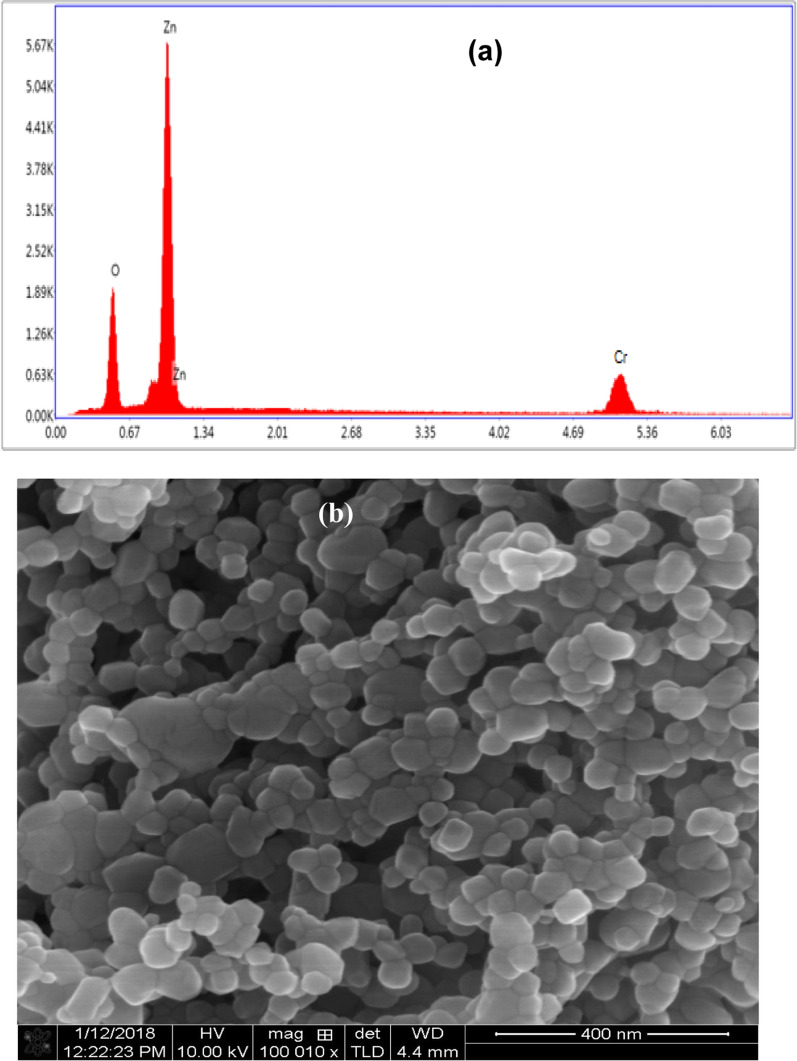
**a** EDX spectrum, **b** SEM micrographs, and **c** TEM image of the ZnCrO nanoparticles annealed at 450 °C

Here $\uprho$ is the density equal to 5.58 g/cm^3^ for zinc chromium oxide. The XRD parameters were calculated and summarized in Table 1. As tabulated, ZnCrO NPs exhibited small size approximately equal to 36 nm and a relatively large specific surface area attributed to generate more charge carriers by chromium ions replacement. Furthermore, the chemical composition and purity of the synthesized nanomaterial were investigated using EDX spectroscopy, Fig. 2a. As observed, the spectrum composed of the main elements Zn, Cr, and O only with atomic percent (at.%) 58.67, 8.86, and 32.47 respectively, which is consistent with the starting composition. The presence of chromium (Cr) in the spectrum clearly supports the successful integration chemical reaction and formation of zinc chromium oxide compound with pure phase [34]. For further microstructure analysis, SEM micrograph was done to visualize the surface topology and shape of the nanoparticles. As depicted in Fig. 2b, the particles are distributed in nanosphere/hexagonal like structures, where some of them agglomerated together [30, 34]. Besides, the mean size of the nanospheres was estimated from the TEM image to be around 25 nm (Fig. 2c) [35, 36].

**Table 1:** Crystallographic parameters of Zn_1−x_Cr_x_O nanostructure

Metal oxide NPs	D (nm)		$\upvarepsilon \times 10^{ - 4}$	δ × 10^–4^ (nm^−2^)	Sa × 10^5^ (cm^2^/g)
	Scherer	W–H			
Zn_1−x_Cr_x_O	33.30	36.00	11.00	8.00	3.00

#### Optical properties of Cr-doped ZnO thin film

**Fig. 3:**
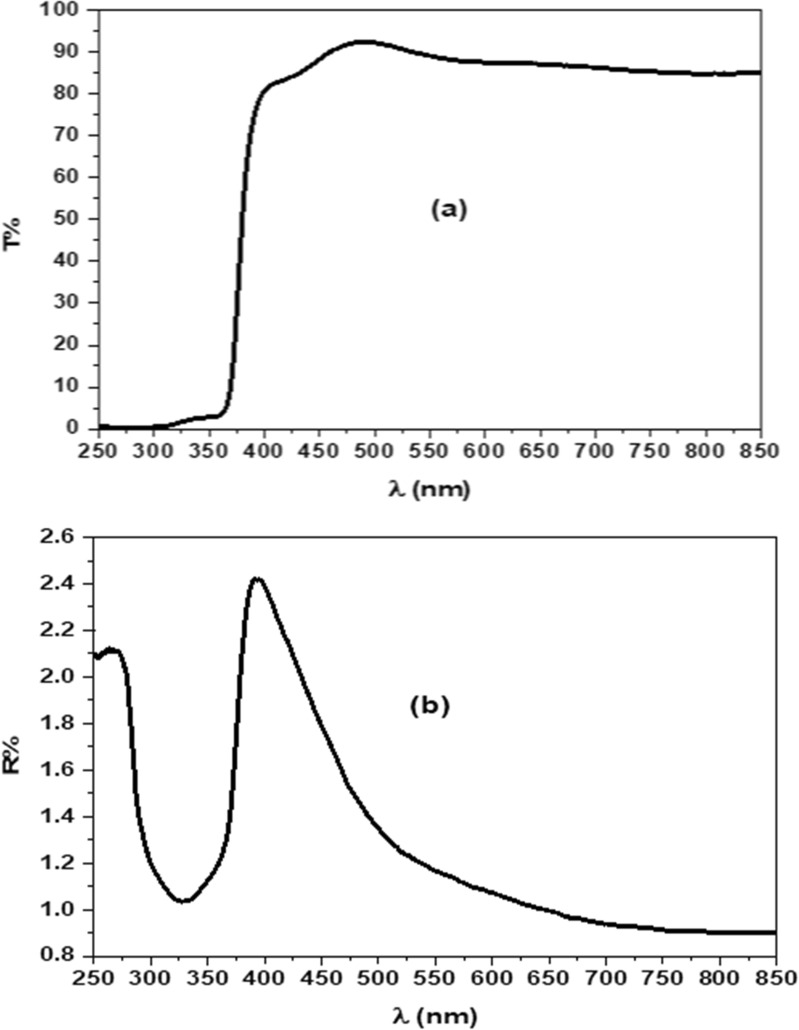
**a** Transmittance spectrum (T%) and **b** reflectance spectrum R%) of Zn_1−x_Cr_x_O thin film

Figure 3a describes the optical transmittance (T%) of ZnCrO thin film through the wavelength range of 250–850 nm. The film exhibited high transmittance above 85% within the visible region of a sharp absorption edge detected at 380 nm associated with electron interband transitions from the valence band to the conduction band [37, 38]. Further, the optical reflectance (R%) displayed valleys and peaks inside the UV region and near the visible spectrum related to the optical-electron interactions, (Fig. 3b). The absorption coefficient $\left( {\upalpha } \right)$ of was calculated by the equation described as [39, 40]:

5 $$\upalpha = \frac{1}{{\text{d}}}\ln \left[ {\frac{{\left( {1 - {\text{R}}^{2} } \right)}}{{2{\text{T}}}} + \sqrt {\frac{{\left( {1 - {\text{R}}} \right)}}{{4{\text{T}}^{2} }} + {\text{R}}^{2} } } \right].$$

**Fig. 4:**
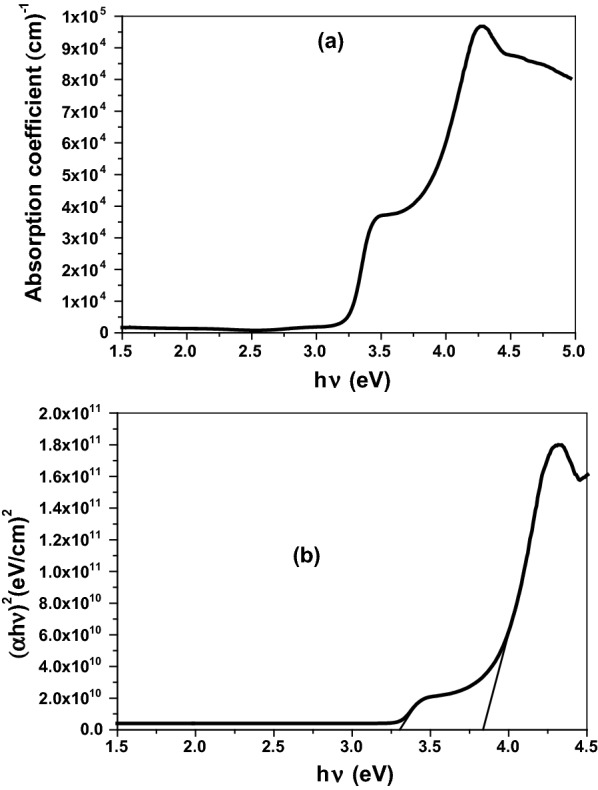
**a** Optical absorption coefficient and **b** (αhν) 2 versus hν plot for Zn_1−x_Cr_x_O thin film

The absorption coefficient $(\upalpha )$ versus photon energy $({\text{h}}\upnu )$ is presented in Fig. 4a. As shown, the film of a weak absorption peak in the UV spectrum while has a strong peak inside the visible region suggesting the strong light absorption and an increase of free charge carriers [40, 41]. The energy gap $\left( {{\text{E}}_{{{\text{g}} }} } \right)$ is very important parameter in the semiconductor materials and transparent nanometal oxides was determined from $\left( {\upalpha {\text{h}}\upnu } \right)^{2}$ against $({\text{h}}\upnu )$ plot (Fig. 4b) according to Tauc’s relation expressed as [40, 41]:

6 $$\upalpha = \frac{{\text{A}}}{{{\text{h}}\upnu }} \left( {{\text{h}}\upnu - {\text{E}}_{{\text{g}}} } \right)^{{\text{n}}} .$$

A is independent constant and n = 1/2 for direct band gap. By extrapolating the linear portion to $\left( {\upalpha {\text{h}}\upnu } \right)^{2}$ = 0, the energy gap of the fabricated thin film was calculated within the visible spectrum in the range 3.307–3.840 eV [42, 43]. The observed high electron transitions and the reduction in the band gap by doping chromium ions results in the surface defects and releasing free Zn^2+^ and Cr^3+^ [11]. In addition to generating reactive oxygen species (ROS) which strongly impact on the disturbance of cellular equilibrium and cell causing locust death.

#### Ultrastructure of the fat body of 5th instar nymphs of *S. gregaria*

**Fig. 5:**
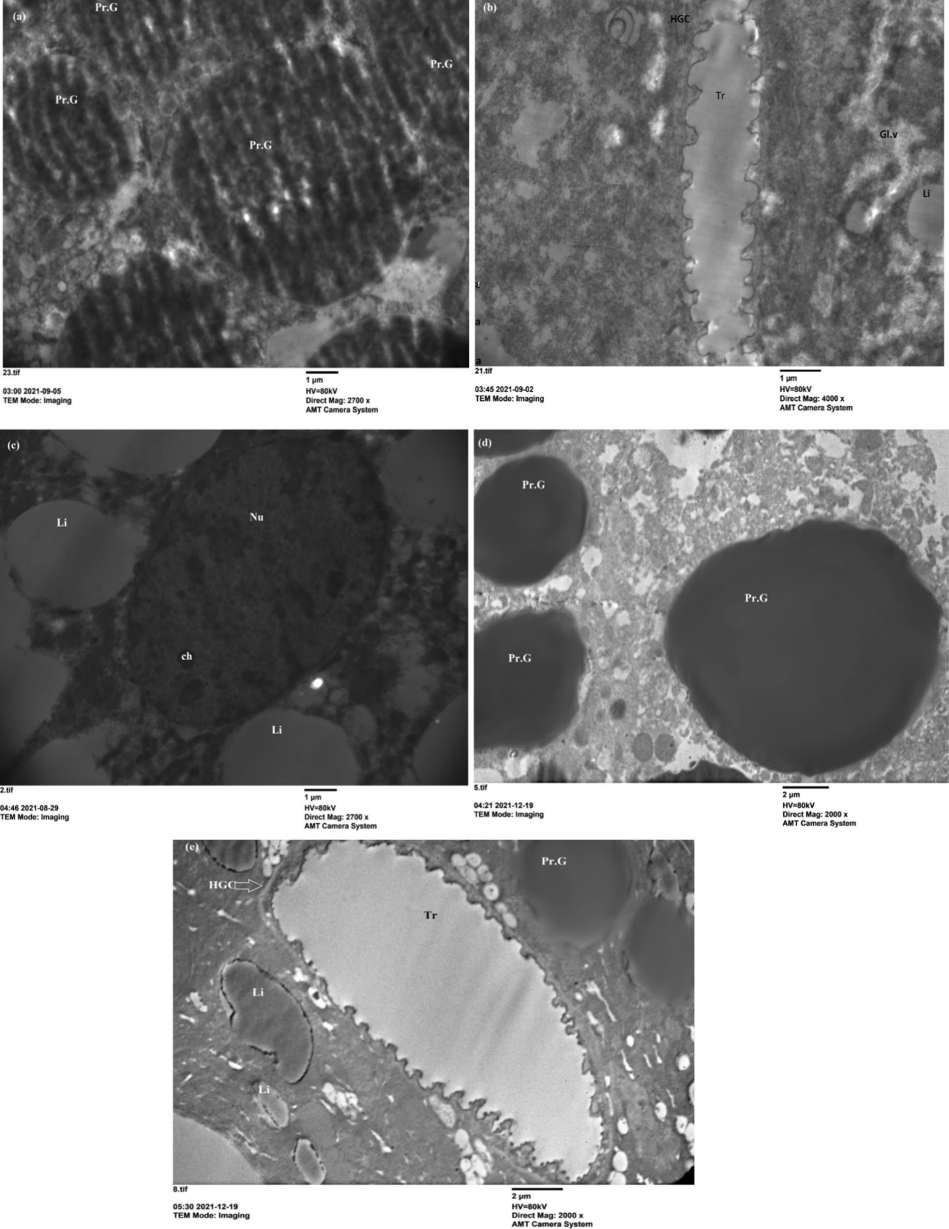
Electron microscope section of fat body cells of untreated 5th instar nymphs of *S. gregaria*. **a**–**c** View of fat body cells showing the general structure of the tissue on 3rd day, protein granules (Pr. G) (×2700), glycogen vacuole (Gl V), haemoglobin cells pierced with the trachea (Tr), lipid droplets (×4000X), nucleus (Nu) with chromatin (ch), lipid droplets (Li) (×2700). **d** Shows protein granules (Pr. G) (×2000) on the 5th day. **e** Protein granules (Pr. G), haemoglobin cells (HGC) pierced by the trachea (×2000) on the 7th day

The fat body has vital roles in the life of insects. It is involved in multiple metabolic functions. One of these functions is to store and release energy in response to the energy demands of the insect [44]. Transmission electron microscopy of the untreated 5th instar nymphs showed that the peripheral fat body cells contained protein granules (PrG) as crystalline storage granules (Fig. 5a, d). The mitochondria (mt) were oval, and the endoplasmic reticulum had the appearance of regular strings and bears ribosomes forming a rough endoplasmic reticulum (rer). The nucleus (Nu) was spherical and possessed chromatin clumps (Ch), which were sporadically scattered throughout the nucleus (Fig. 5c). Haemoglobin cells (HGCs) were pierced by trachea (Tr) and lipid droplets (Li) (Fig. 5b, e). Insect adipocytes store a large amount of lipid reserves as cytoplasmic lipid droplets. Lipid metabolism is necessary for growth and reproduction and provides the energy needed during extended nonfeeding periods [44–47]. The previous literatures described the ultrastructure of the fat body as the lipids are deposited as small drops or in large vacuoles that may occupy most of the cytoplasm. The proteins form electron dense granules of variable sizes and shapes, in some cases appearing crystallized [47–52].

**Fig. 6:**
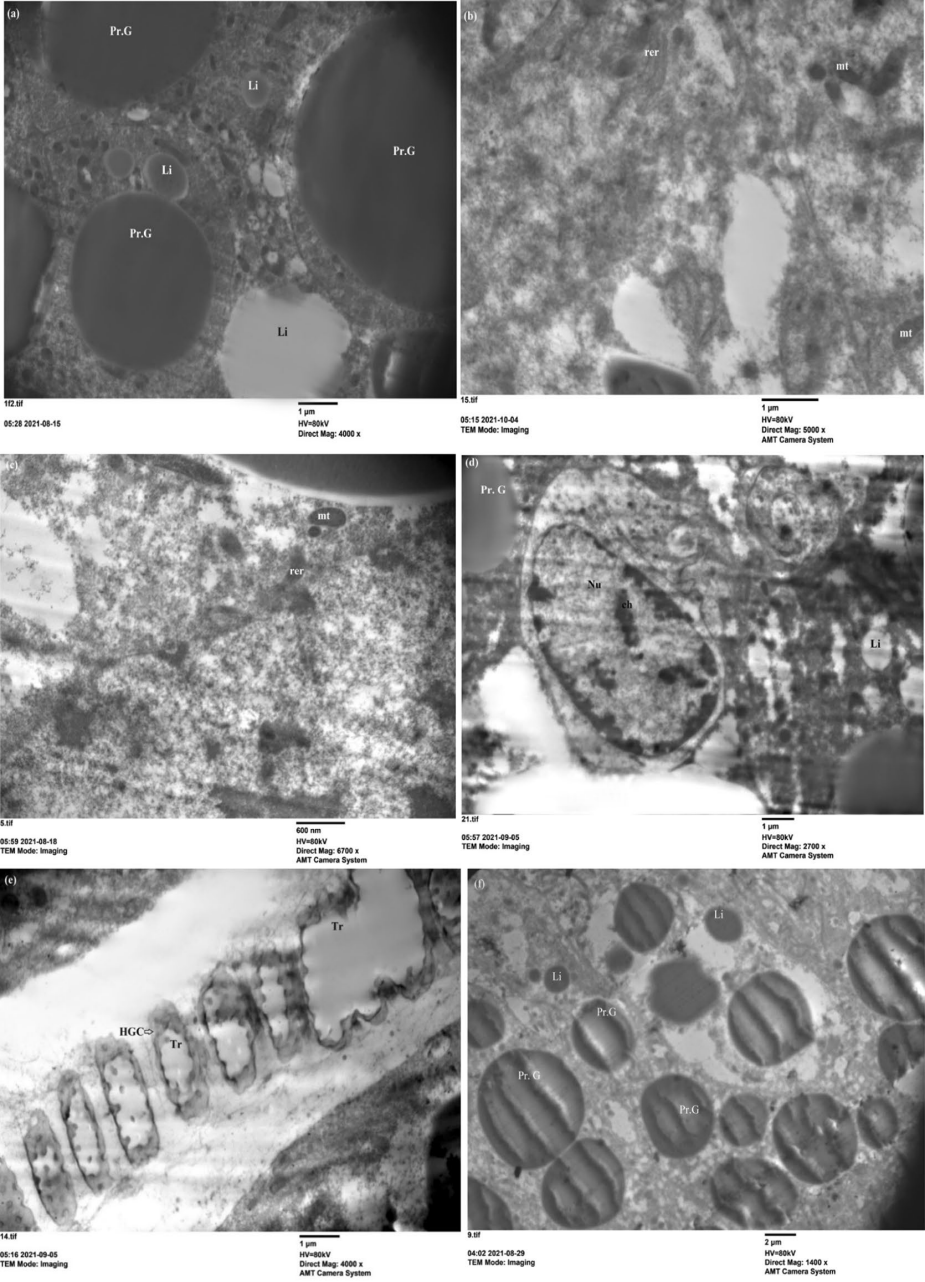

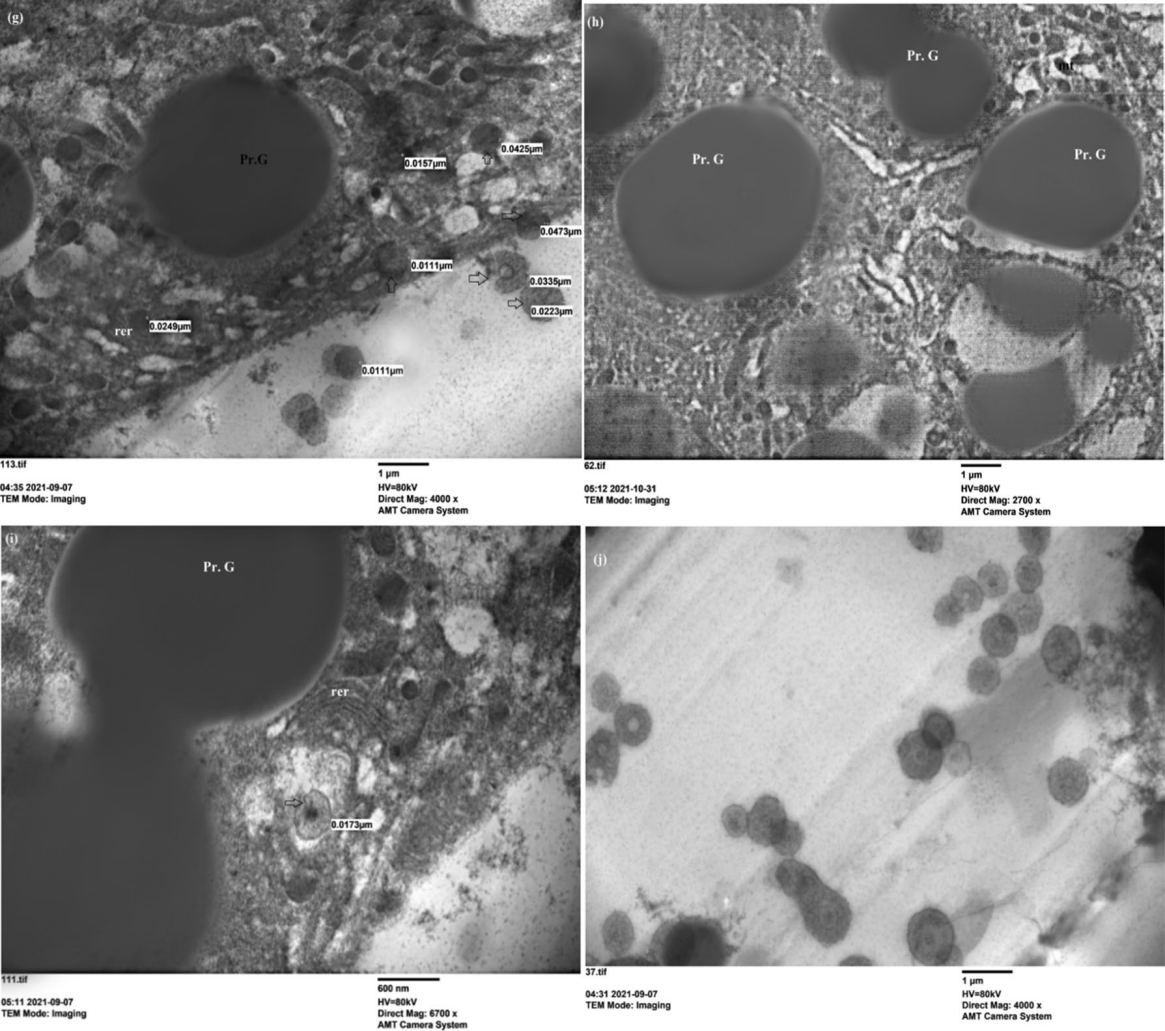
Electron microscope section of fat body cells of treated 5th instar nymphs of *S. gregaria* sprayed with 2 mg of zinc chromium oxide. **a**, **b** View of fat body cells showing protein granules (Pr. G), lipid droplets (Li) (×4000); rough endoplasmic reticulum (rer), mitochondria (mt) (×5000) on the 3rd day post treatment. **c**–**e** Show rough endoplasmic reticulum (rer), mitochondria (mt) (×6700); nucleus (Nu) with chromatin clumps (ch), lipid droplets (Li) (×2700); haemoglobin cells pierced with the trachea (Tr) (×4000) on the 5th day post treatment. **f** Protein granules (Pr. G), lipid droplets (Li) (×1400) on the 7th day post treatment. **g**–**i** Show Protein granules (Pr. G) and crystals of nanoparticles (4000, 2700, ×6700) on the 7th day post treatment. **j** Crystals of ZnCrO around the tissues of fat body (×4000) on the 7th day post treatment

On the other hand, the treatment nymphs with 2 mg of ZnCrO NPs declared no effect on the fat body organelles at 3rd day of post treatment (Fig. 6a, b). But, at 5th day post treatment mitochondria was swollen and dilation of cristerana of rough endoplasmic reticulum were observed (Fig. 6c). The malformed nucleus has peripheral chromatin and haemoglobin cells (HGCs) pierced by destroyed trachea which affected the respiration process (Fig. 6d, e). After 7 days of spraying 2 mg of ZnCrO, numerous cytolyosomes that digest cytoplasmic organelles were illustrated and were indicative of cytoplasmic deterioration and leads to cell death [53] and protein granules were shrinked  and effected on protein metabolism (Fig. 6f). Crystals of nanoparticles showed around protein granules (Fig. 6g–j). The mean average size of ZnCrO observed between the protein granules of the fat body of 5th instar nymphs was 26 nm. This agreed with the size of the particles used.

**Fig. 7:**
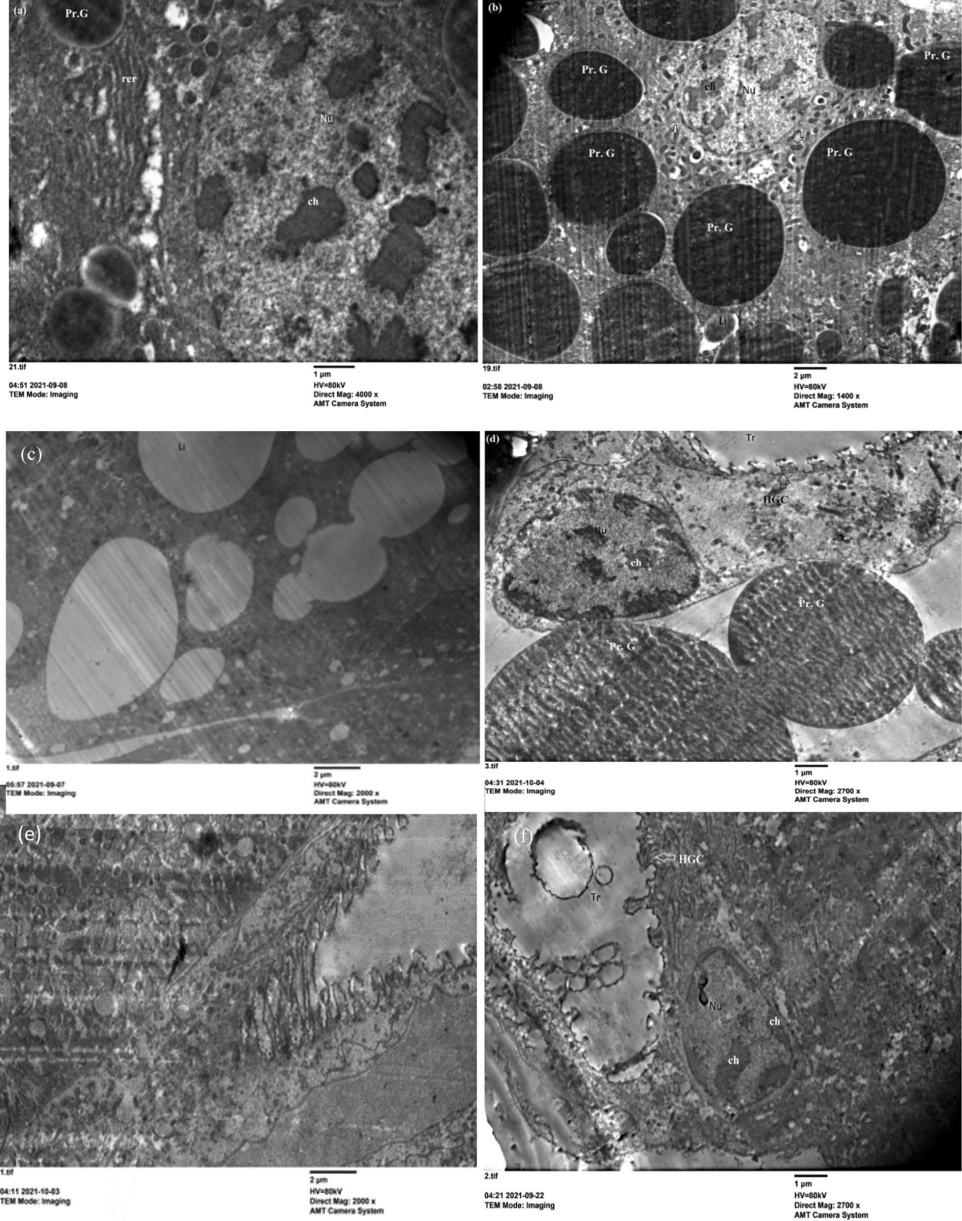
Electron microscope section of fat body cells of treated 5th instar nymphs of *S. gregaria* sprayed with 1 mg of zinc chromium oxide. **a**–**c** Show rough endoplasmic reticulum (rer), nucleus (Nu) with chromatin clumps (ch) (×4000); protein granules (Pr. G) and nucleus (Nu) with chromatin clumps (ch) (×1400); lipid droplets (Li) (×2000) on the 3rd day post treatment. **d**, **e** Protein granules (Pr. G), nucleus (Nu) with chromatin clumps (ch), haemoglobin cells (HGC) pierced with the trachea (Tr) (×2700); haemoglobin cells (HGC) pierced with trachea (Tr) (×2000) on the 5th day post treatment. **f** Haemoglobin cells (HGC) pierced with the trachea (Tr) (×2700) on the 7th day post treatment

**Fig. 8:**
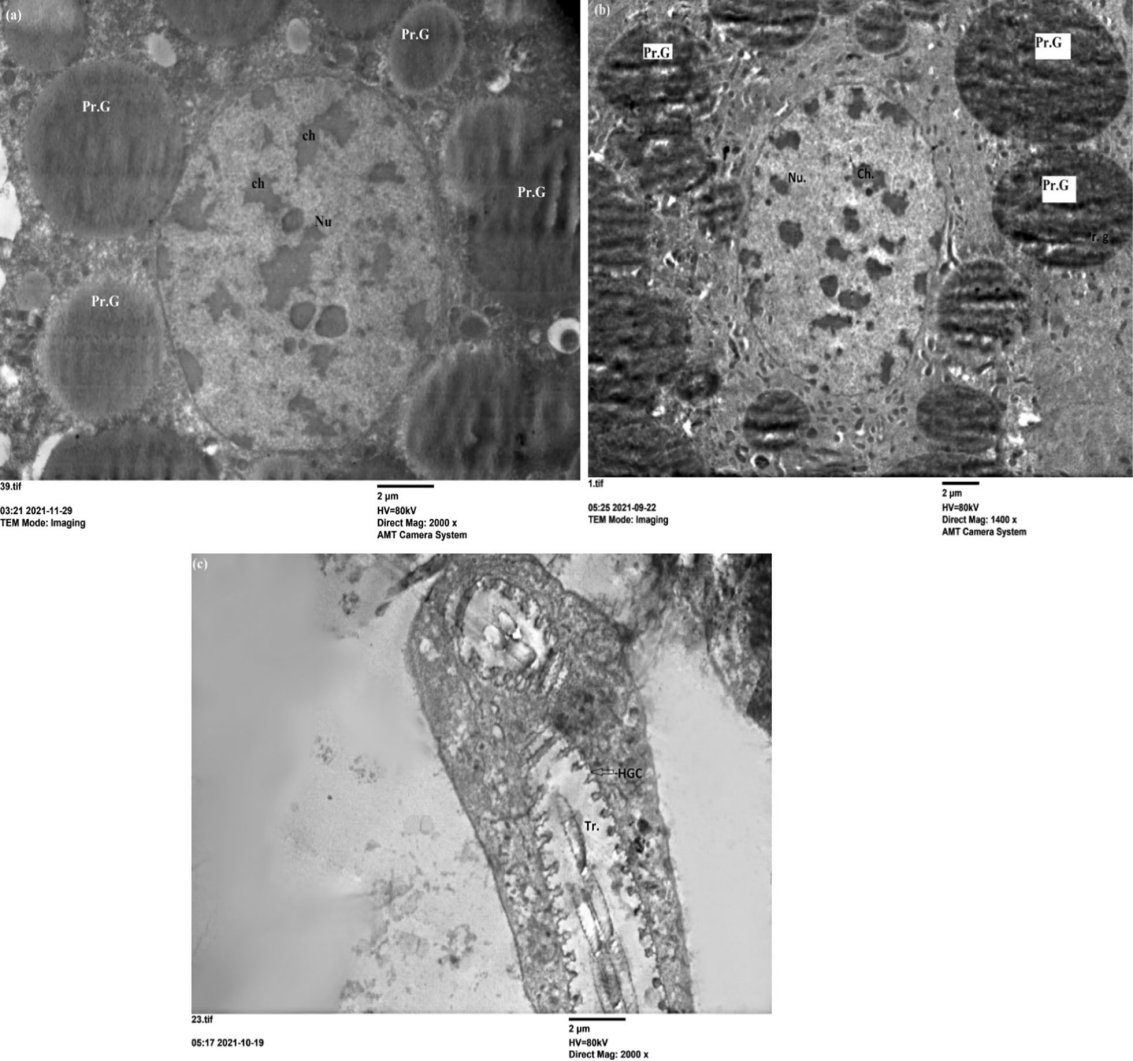
Electron microscope section of fat body cells of treated 5th instar nymphs of *S. gregaria* sprayed with 0.150 mg of on zinc chromium oxide. **a** Shows protein granules (Pr. G), nucleus (Nu) with chromatin clumps (ch) (×2000) on the 3rd day post treatment. **b** Protein granules (Pr. G), nucleus (Nu) with chromatin clumps (ch) (×1400) on the 5th day post treatment. **c** Haemoglobin cells (HGC) pierced with the trachea (Tr) (×2000) on the 7th day post treatment

The effect of nanoparticles decreased when sprayed with 1 mg of ZnCrO compared to the high concentration. The protein granules, lipid droplets and nucleus appeared in normal shape on the 3rd day post treatment with nanoparticles (Fig. 7a–c). Although at 5th and 7th days post treatment abnormal nucleus with crowded chromatin (Fig. 7d) and malformed trachea (Fig. 7e, f) were detected. However, the lowest concentration (0.150 mg) NPs showed normal protein granules, nuclei, and trachea (Fig. 8a–c). This study demonstrated that the nanoparticles caused structural damage in the fat body cells of immature stages and could alter the development of locusts. These changes cause the malfunction of fat body cells, particularly at a high concentration, leading to nymphal death. Meng et al. [54] reported that *Bombyx mori* (Lepidoptera: Bombycidae) showed low resistance against Ag NPs to oxidative stress, affected cell apoptosis, and induced cell necrosis by regulating related protein metabolism and metabolic pathways. Ag NPs can also reduce the ability of silkworms to withstand oxidative stress and interfere with programmed cell death.

Furthermore, the fat body cell malformations in this work is agreed with those described in the fat body cells of different insects exposed to azadiractin, neem oil, plant-derived, and insecticides such as swollen mitochondria in the cristolysis process, dilated cisternae of the rough endoplasmic reticulum, the fusion of lipid droplets, and dilation of the perinucleus [55–61]. Moreover, although the fat body was not directly exposed to ZnCrO nanoparticles by injection, it is a good target in ecotoxicological studies.

### Conclusions

Cr doped ZnO (10 wt%) nanopowder was successfully synthesized with cost-effective chemical co-precipitation technique. The XRD pattern indicated that the nanomaterial of polycrystalline structure in pure phase in which chromium ions incorporated into the host ZnO framework. The EDX spectrum confirmed the presence of Cr^3+^ inside ZnO lattice structure and production of ZnCrO compound. The crystallographic parameters $\left( {{\text{D}}, \varepsilon , \delta ,\rho } \right)$ were calculated showing that the nanoparticles of small grain size ~ 36 nm. The surface topological aspects of ZnCrO revealed that the NPs of spherical–hexagonal shapes with large surface to volume area. The optical properties displayed the prepared thin film of high transparency in the visible region and wide energy gap. The fat body is a good target organ in ecotoxicological studies by providing the details of injuries and cellular response. However, it was not directly exposed to zinc chromium oxide NPs by intake or spraying.

## Data Availability

The datasets used and/or analyzed during the current study available from the corresponding author on reasonable request.

## Abbreviations

NPs: Nanoparticles
XRD: X-ray diffraction
EDX: Energy dispersive X-ray spectroscopy
SEM: Scanning electron microscopy
TEM: Transmission electron microscopy
HGCs: Haemoglobin cells
Pr. G: Protein granules
mt: Mitochondria
Li: Lipid droplets
GlV: Glycogen vacuole
Tr: Trachea
Nu: Nucleus
rer: Rough endoplasmic reticulum
Ch: Chromatin clumps
